# Successfully Treated Complications After TAVI Implantation - Single Center Experience

**DOI:** 10.1186/1749-8090-10-S1-A50

**Published:** 2015-12-16

**Authors:** Dimitar Petkov, Dimitar Kyuchukov, Peyo Simeonov, Diana Trendafilova, Julia Jorgova, Gencho Nachev

**Affiliations:** 1 Department of Cardiac Surgery, "St. Ekaterina" University Hospital, Sofia, Bulgaria 1431

## Background/Introduction

TAVI is proven as a beneficial procedure in high risk patients with severe aortic stenosis.

## Aims/Objectives

Our hospital is the only center in Bulgaria, which implants TAVI. Here we report some rare complications of TAVI and their successful management.

## Method

Between 2008 and 2015 a total number of 67 TAVI were implanted in 64 patients in our center. The average age of the patients was 76 years (between 64 and 85). There were 41 female and 23 male patients. The average EuroSCORE was 27.31% (between 13.2% and 47.4%), while the average STS score was 9.42% (between 4.1% and 24.2%).

## Results

In five patients we had intraprocedural complications, which require attention.

1) Localized aortic root rupture with formation of subaortic intracardiac fistula, after transaortic implantation. Since the fistula was blind-ended no further interventions were made right after the complication was detected. However on 14th postprocedural hour another angiography was performed, because of signs of STEMI. It revealed compression of RCx by the fistula. Balloon dilatation was performed with excellent result.

2) We experienced intraprocedural partial valve dislocation in LV after TAVI implantation with subclavian access. After unsuccessful attempt for repositioning the valve a second valve was implanted in valve-in-valve fashion in order to stabilize the displaced one. The result was excellent.

3) Total intraprocedural valve dislocation into the LV after transapical implantation. Since the valve was in the LV all attempt for repositioning were unsuccessful. Finally it was removed through the LV apex, without using of extracorporeal circulation. A subsequent successful transfemoral implantation was performed, during the same procedure.

4) Two patients had LV perforation complicated with huge pericardial effusion as a result of stiff wire manipulation during transfemoral approach. Urgently these patients received sternotomy for LV suture.

All five patients recovered uneventfully and were discharged home.

## Discussion/Conclusion

Here we report five successfully managed intraprocedural complications of TAVI. Complication awareness is crucial for their early detection and successful treatment. Increasing the experience will decrease the overall number of complications.

